# Blood Lead Level and Handgrip Strength in Preadolescent Polish Schoolchildren

**DOI:** 10.3390/toxics10110646

**Published:** 2022-10-27

**Authors:** Natalia Nowak-Szczepanska, Aleksandra Gomula, Anna Sebastjan, Zofia Ignasiak, Robert M. Malina, Sławomir Kozieł

**Affiliations:** 1 Department of Anthropology, Hirszfeld Institute of Immunology and Experimental Therapy, Polish Academy of Sciences, 53-114 Wroclaw, Poland; 2 Department of Biostructure, Wroclaw University of Health and Sport Sciences, 51-612 Wroclaw, Poland; 3 Department of Kinesiology and Health Education, University of Texas at Austin, Austin, TX 78712, USA

**Keywords:** lead, grip strength, preadolescent children

## Abstract

Environmental pollutions, particularly toxic elements such as lead, are among the most significant factors affecting the growth and functional development of children. The aim of this study was to evaluate the effect of blood lead levels on handgrip strength (HGS) in urban children resident in the Copper Basin of Lower Silesia, Poland, controlling for the effects of chronological age, body size and socioeconomic status. The study included 165 boys (9–11 years of age) and 79 girls (9–10 years of age) from Polkowice town. Anthropometric measurements involved height, body mass and grip strength of the left and right hands. Maternal education was a proxy for socioeconomic status. Based on the median value of blood lead level (3.10 µg/dL), the two groups-below and above/equal median value-were defined. Analysis of covariance revealed that age (all *p* < 0.001), sex (at least *p* < 0.01), BMI (all *p* < 0.001), and blood lead level (at least *p* < 0.05) had a significant effect on the three indicators of HGS (right hand, left hand, average), while the level of maternal education did not significantly affect HGS (*p* > 0.05). The results of this study indicate a potentially negative effect of elevated blood lead level on grip strength in preadolescent children, irrespective of sex.

## 1. Introduction

Handgrip strength (HGS) is a commonly used indicator of overall muscular strength in children, adolescents and adults [1]. Muscular strength is an important component of physical fitness, both health- and performance-related fitness, and is also related to physical activity [2,3,4]. Along with metabolic function, grip strength may also play important role in health and disease [5]. From an epidemiological perspective, HGS may also have prognostic value as a potential indicator of risk of all-cause and cardiovascular mortality or morbidity in adults (older than 40 years of age) [6]. Grip strength is recorded as the maximum voluntary contraction of the grip flexor muscles and has a long tradition in studies of growth and performance [7]. Motivation is a factor in testing grip strength as perception of maximal effort varies among individuals and likely with age. Sex differences in grip strength are negligible in childhood and are established during pubertal maturation and the growth spurt [8]. Males have higher grip strength than females beginning from adolescence. Three periods in development of grip strength are suggested: increase up to peak in early adulthood, maintenance during midlife, and decrease from midlife onward, with sharp increase in weak grip strength with age [9]. Grip strength is related to body size and also to variation in biological maturity status in boys more so than in girls. Grip and other indicators of muscular strength also have their own growth spurts, which occur, on average, after the growth spurt in height and are more consistent with the growth spurt in body weight [8].

Environmental pollutants are significant factors affecting the growth and functional development of children and youth. More specifically, toxics elements, such as lead, arsenic, cadmium or mercury, may interfere substantially with prenatal and postnatal growth [10,11,12]. Lead exposure among growing children may also have a negative influence on neurodevelopment [13], somatic growth [12] and maturational timing [14,15]. Recent auxological studies have showed reduced growth in height, smaller head circumference, delayed puberty, including later menarche, and impaired nutritional status in children with high blood lead levels [16,17,18]. Although focus is commonly on industrial environmental pollutants, observations of indigenous children in Canada (Inuit, northern Quebec) noted a negative influence of several pollutants, including lead, associated with consumption of contaminated seafood and motor coordination [19,20]. Of interest, in a national sample of U.S. adults 20–79 years, elevated blood lead levels were associated with a marked reduction in the grip strength of women but not of men [21], while prenatal lead exposure revealed no adverse effect on motor skills at age 7 [22].

It should also be noted that a variety of other factors influence growth in body size, muscle mass and muscular strength during childhood and adolescence. Absolute muscular strength, for example, was reduced in children and adolescents in populations with a history of chronic undernutrition; when expressed per unit body mass, however, strength was similar to that in better-nourished populations [23,24]. Similarly, among Polish youth from the rural areas of the Copper Basin exposed to lead [25], the reduction in grip strength was commensurate with body size in both boys and girls [26]. However, the effect of blood lead level on measures of physical performance in this sample surveyed in 1995 was rather indirect and relatively small [26]. The present study is an extension of the earlier analyses which focused on rural youth [25,26]. It specifically considers relationship between blood lead level and grip strength in a sample of Polish urban children, examined in Polkowice town in 1996. Specifically, this survey aims to consider the effect of blood lead level on grip strength in urban youth resident in the Copper Basin of Lower Silesia, Poland, statistically controlling for the effects of chronological age, body size and socioeconomic status (SES).

## 2. Materials and Methods

### 2.1. Participants

The study included 165 boys 9–11 years old and 79 girls 9–10 years old. The respective age ranges were selected considering the level of biological development, generally accepted as pre-adolescent [27]. Adolescent children were excluded from the study due to the differences in developmental tempo, which could significantly affect examined biological features, as maturation is related to significant changes in body size at different rates. The study was conducted in all four primary schools in Polkowice, a town located in the central part of the Copper Basin, Lower Silesia, Poland, with a population of over 22,000 inhabitants. In the mid-1990s, the municipality of Polkowice was one of the richest and well-developed regions in Poland due to investment in socio-economic development associated with a highly developed mining industry [28].

The children were part of comprehensive study of the anthropometric and functional characteristics of schoolchildren aged 9–11 years in 1996. Parents provided written consent for the participation of their children in the research project at the last meeting of parents and teachers in a given school year. These consents were passed on by the school headmaster to the research manager. Then, the research schedule was established and the study was carried out in the second half of September during subsequent school year. Parents willingly consented to the participation of their children in the study, unless the children had any illnesses. Final response rate was approx. 80%. This study was conducted in accordance with the recommendations of the Helsinki Declaration, and was approved by the Education Office of the City Hall of Polkowice and the School Headmasters of the four schools in Polkowice. The study was also approved by the ethics committee of the University School of Physical Education in Wroclaw.

### 2.2. Measurements

Anthropometric measurements were performed referring to the standard measuring procedures developed by Martin and Saller [29]. Height, body mass and grip strength of the left and right hands were measured in children in all investigated schools by the staff of the Department of Biostructure at the University School of Physical Education in Wrocław, Poland. Height was measured to the nearest 1 mm with an anthropometer (Swiss company GPM Instruments, Gneupel, Switzerland). Body mass was measured to the nearest 0.1 kg with a medical scale. The children were examined in light sportswear without footwear. The body mass index (BMI, weight/height^2^; kg/m^2^) was calculated. Grip strength of the right and left hands was measured to the nearest 1.0 kg with adjustable dynamometer (Jamar, Sammons Preston Rolyan, Bolingbrook, IL, USA). While in a standing position, the child was instructed to grasp the dynamometer as hard as possible, giving a maximum effort. The measurement was performed twice for each hand. The higher result for each hand was retrained for analysis.

Information on maternal level of completed education (elementary, trade, college, university), commonly used as one of the best indicators of SES, was obtained with a questionnaire completed by the parents. In our study sample, 15.8% of mothers had elementary education, 37.5%-trade, 40.4%-college, and 6.3%-university.

Lead levels were estimated from blood samples taken after a fast in the office of the school nurse at the respective schools by the qualified personnel of the Foundation for Children from the Copper Basin (Legnica, Poland). The samples were taken through an intravenous Vacutainer tube. Right after collection, the samples were directly transported in the special refrigerators to the laboratory and tested on the same day for lead concentration using atomic absorption spectrometry in a Hitachi Z-8200 graphite-tube furnace with Zeeman background correction; (this is a method for rapid determination of lead in blood; for more details, see: [30]). The analyses were performed with standard laboratory practices and appropriate standards (Nycomed, Sweden) in the nationally accredited Heavy Metals Toxicology Laboratory of the Foundation for Children from the Copper Basin (Legnica, Poland). The minimal detectable lead level was <0.1 µg/dL. The laboratory procedures included constant quality control [31].

### 2.3. Statistics

Sex differences in the three indicators of grip strength (right, left, average of the two) and mean blood lead level were evaluated with the Mann–Whitney U-test. The median blood lead level for the study sample was 3.10 µg/dL. Two groups were subsequently formed relative to the median: <3.10 µg/dL and ≥3.10 µg/dL. Analysis of covariance (General Linear Model) was implemented to assess the effect of blood lead level category (as an independent factor) on left, right and average HGS (as dependent variables), taking into account mother’s education (elementary, trade, college, university) and sex as other independent factors as well as children’s age and BMI as covariates. Effect of interactions between blood lead level and maternal education as well as BMI on HGS was also investigated. All analyses were done using Statistica 13.1 (TIBCO Software Inc., Palo Alto, CA, USA).

## 3. Results

Descriptive statistics for blood lead level and measures of grip strength in boys and girls are summarized in Table 1. Boys and girls did not differ significantly in blood lead level, although boys had slightly higher mean values and wider range of blood lead levels. On the other hand, right and left grip strength as well as average grip strength was significantly higher in boys compared to girls (*p* < 0.001).

**Figure 1:**
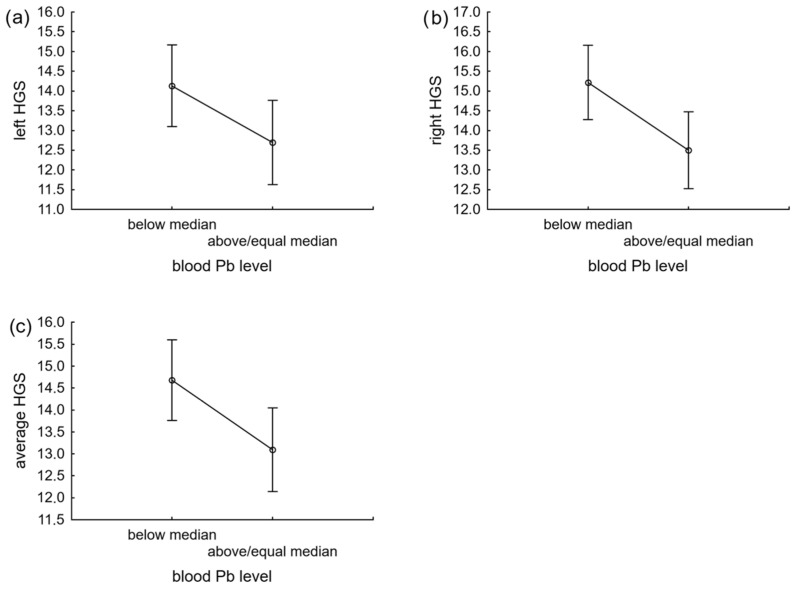
Differences in (**a**) left, (**b**) right and (**c**) average HGS (means and CI) between blood lead level groups (below or equal and above the median value = 3.10 μg/dL). All children with blood lead level below the group median had a significantly higher level of strength of the left and right hands as well as average HGS, irrespective of sex.

Results of the analysis of covariance are summarized in Table 2. Age (*p* < 0.001), sex (at least *p* < 0.01), BMI (*p* < 0.001), and blood lead level (at least *p* < 0.05) had a significant effect on the three indicators of HGS, while level of education of the mother did not significantly affect HGS (*p* > 0.05). The effect size (interpreted by the partial η^2^ value) were the highest for age and the lowest for blood lead level; both were statistically significant (Table 2). The interactions between blood lead level and sex, maternal education as well as BMI were not significant (*p* > 0.05). Thus, the results of the GLM model for equal slopes were presented. It should be highlighted that Polkowice was one of the richest and relatively homogenous (in terms of SES) places in this region. Thus, potential socioeconomic differences might not have been so pronounced and, consequently, have not differentiated visibly the physical development of children, as reflected in non-significant effect of interaction between blood Pb level and maternal education on HGS. All children with blood lead level below the group median had a significantly higher level of strength of the left and right hands and also average HGS, irrespective of sex (Figure 1).

## 4. Discussion

This research is one of the first studies that show relationship between blood lead concentration and the level of muscle strength assessed by hand grip strength in preadolescent urban children. The children were born and reared in a region with relatively constant levels of environmental pollution associated with heavy metals. Although the Polkowice municipality is one of the wealthier regions in Poland due to the active mining industry and the population is rather uniformly better-off socioeconomically, the results of the present study noted significantly reduced levels of grip strength in preadolescent children with blood lead levels that were equal to or above the median compared to peers with blood levels below the group median, regardless of sex.

Recent research has shown that higher blood lead levels in children are associated with indicators of nutritional status that are lower compared to peers with lower blood lead levels [17]. Since the development of muscle mass, and in turn muscular strength, is associated with nutritional status, particularly in childhood, nutritional deficits can lead to negative effects on muscular development. Exposure to lead in the environment of the Polkowice region has likely had an influence on the growth of children, beginning prenatally and continuing through infancy into childhood. As such, it is likely that the development of muscle mass and muscular strength may have been affected by the higher concentrations of lead beginning early in life. Nevertheless, it should be also noted that the genetic factors can significantly affect the primordial number of muscle fibres in utero [32]. Thus, the effect of environmental influences may be, to some extent, limited in this context, since blood Pb level in our study sample had much lower effect size on HGS than children’s sex, age or BMI. On the other hand, SES as reflected in mother’s education did not significantly affect HGS.

An earlier study in 1995 considered the association between blood lead level and several indicators of physical fitness, including grip strength, among children and youth 7–15 years of age in several rural schools in the Legnica-Głogów region of the Copper Basin [26]. The effect of blood lead level on grip strength (sum of right and left hands) in the rural boys was influenced by reduced body size, but the effect was statistically negligible; the corresponding effect of body size on grip strength in the rural girls was stronger [26]. Allowing for the small effects of blood lead level on grip strength, the overall results suggested no direct association between blood lead concentration and the other indicators of fitness considered (sit-ups, flexed arm hang, standing long jump, shuttle run, medicine ball throw, and speed of limb movement). The results suggested that blood lead level indirectly affected the indicators of fitness through its negative effect on growth in body size. Note, however, blood lead levels in the rural children and youth [25] were, on average, higher than those in the present sample from the Polkowice town. Nevertheless, in the present study conducted in Polkowice town, the interaction between blood lead level and sex, was not significant and results of analysis of covariance suggested that a higher blood lead level was associated with reduced grip strength, irrespective of sex.

Many studies have shown that heavy metals, particularly lead, have an adverse effect on human development and health [25,33,34,35]. It is possible that permanent lead exposure among children in the Copper Basin, which began early in development and continued postnatally, had a negative influence not only on linear growth, nutritional status and maturation [17,18,25,36], but could also affect muscle development leading to the impairment of muscular strength measured by HGS. Implications of exposure to heavy metals on muscular development and strength (and, specifically, HGS) have not received much attention in the literature. Previous study on children from rural areas of Copper Basin in 1995 showed inverse, but indirect (mediated by body size), association of blood Pb level with HGS in girls, while in boys this relationship was rather weak (see: [26]). With respect to the possible influence of heavy metals on muscle strength development, there is one study on animal model showing the negative effects of mercury vapor inhalation on the level of muscle strength in mice [37]. Several clinical studies noted an association between heavy metals exposure, such as mercury or lead, and its potentially toxic effects on motor neurons, and by inference a potential relationship between heavy metals intoxication and amyotrophic lateral sclerosis (ALS), a neurodegenerative disease associated with muscular atrophy and weakness [38,39]. The biological processes underlying these associations have not been explored in detail and the underlying physiological mechanisms of the potential relationship between lead exposure and muscular strength and functioning remains to be established.

The potential negative influence of elevated blood lead levels on the nutritional status of children and youth living in the Copper Basin [17] merits further study in the context of muscle strength impairment, since chronic undernutrition may be related to lower levels of muscular strength [40]. Nevertheless, the present results did note an independent effect of blood lead level on grip strength after statistically controlling for the BMI. Further research is also needed to address the biological mechanisms and environmental factors, especially with regard to heavy metal pollution, associated with the muscular development of children. This could support early interventions to help maintain optimal muscle strength in the successive stages of ontogenesis, particularly in children living in lead-contaminated areas.

The present study is not without limitations. Dietary information and information related to lifestyle, specifically patterns of physical activity and physical inactivity (sedentary behavior) were not available. Moreover, information on body composition, specifically fat-free mass, was also not available. However, we did control body mass index as a possible and important effect of body size. Furthermore, we did not examine the level of sex hormones, which are an important factor in the development of muscle and skeletal tissues. Nevertheless, we limited our study group to preadolescent children only, to exclude potential effect of differences in maturational timing. Thus, the study sample could be seen as relatively small, however, the advantage was a similar period of biological development in examined children.

## 5. Conclusions

The results of this study indicate a potentially negative effect of elevated blood lead level on grip strength in preadolescent children, irrespective of sex. The results are generally consistent with previous studies indicating that there is no safe level of exposure to lead since even slightly elevated levels of blood lead (in this study: above 3.10 µg/dL) may negatively influence muscular strength development in children. Further studies are needed to address the possible mechanisms leading to a reduction in muscular tissue and, by inference, muscular strength associated with heavy metal exposure during children’s development.

## Figures and Tables

**Table 1:** Descriptive statistics and results of the Mann–Whitney U-tests for blood lead level and the three indicators of grip strength (left, right, average HGS) in preadolescent boys (9–11 years) and girls (9–10 years) from Polkowice 1996. Correlation between left and right HGS: r_s_ = 0.80, *p* < 0.0001.

	Boys *n* = 165				Girls *n* = 79				
Variables	Mean (SD)	Median	Min	Max	Mean (SD)	Median	Min	Max	U-Test
Pb [μg/dL]	3.70 (2.37)	3.10	0.20	11.80	3.30 (2.18)	2.90	0.50	9.00	Z = 1.28, *p* = 0.20
left HGS	14.92 (5.00)	15.00	1.00	27.00	10.78 (4.69)	11.00	1.00	21.00	Z = 6.11, *p* < 0.001
right HGS	16.06 (4.92)	16.00	1.00	28.00	11.25 (4.23)	12.00	1.00	23.00	Z = 7.17, *p* < 0.001
average HGS	15.40 (4.78)	11.50	0.50	26.50	10.88 (4.45)	15.50	0.50	22.00	Z = 6.90, *p* < 0.001
BMI	17.50 (2.90)	16.55	12.39	27.43	16.55 (2.33)	16.45	13.01	23.33	Z = 2.34, *p* < 0.05

**Table 2:** Results of analysis of covariance for left HGS, right HGS and average HGS as dependent variables and blood level group (below or equal and above the median value = 3.10 μg/dL), maternal education (elementary, trade, college, university) and sex as independent factors and age and BMI as covariates in preadolescent boys (9–11 years) and girls (9–10 years) from Polkowice 1996.

	Left HGS			Right HGS			Average HGS		
Variables	F	*p*	Partial η^2^	F	*p*	Partial η^2^	F	*p*	Partial η^2^
age	29.09	<0.001	0.117	30.06	<0.001	0.120	33.90	<0.001	0.134
sex	8.40	<0.01	0.037	20.14	<0.001	0.084	15.20	<0.001	0.065
BMI	15.58	<0.001	0.066	16.78	<0.001	0.071	18.51	<0.001	0.078
mother’s education	0.56	ns	0.008	0.70	ns	0.009	0.67	ns	0.009
blood Pb level	5.31	<0.05	0.024	9.13	<0.01	0.040	8.05	<0.01	0.035

ns—non significant.

## Data Availability

Restrictions apply to the availability of these data. Data were obtained from Wroclaw University of Health and Sport Sciences and are available from the authors with the permission of Wroclaw University of Health and Sport Sciences.

## References

[1] Wind A.E., Takken T., Helders P.J., Engelbert R.H. (2010). Is grip strength a predictor for total muscle strength in healthy children, adolescents, and young adults?. Eur. J. Pediatr..

[2] Malina R.M. (1975). Anthropometric correlates of strength and motor performance. Exerc. Sport Sci. Rev..

[3] Malina R.M. (2001). Physical activity and fitness: Pathways from childhood to adulthood. Am. J. Hum. Biol..

[4] Strong W.B., Malina R.M., Blimkie C.J., Daniels S.R., Dishman R.K., Gutin B., Hergenroeder A.C., Must A., Nixon P.A., Pivarnik J.M. (2005). Evidence Based Physical Activity for School-Age Youth. J. Pediatr..

[5] Wolfe R.R. (2006). The underappreciated role of muscle in health and disease. Am. J. Clin. Nutr..

[6] Leong D.P., Teo K.K., Rangarajan S., Lopez-Jaramillo P., Avezum A., Orlandini A., Seron P., Ahmed S.H., Rosengren A., Kelishadi R. (2015). Prognostic value of grip strength: Findings from the Prospective Urban Rural Epidemiology (PURE) study. Lancet.

[7] Malina R.M. (1996). Tracking of physical activity and physical fitness across the lifespan. Res. Q. Exerc. Sport.

[8] Malina R.M., Bouchard C., Bar-Or O. (2004). Growth, Maturation and Physical Activity.

[9] Dodds R.M., Syddall H.E., Cooper R., Benzeval M., Deary I.J., Dennison E.M., Der G., Gale C.R., Inskip H.M., Jagger C. (2014). Grip strength across the life course: Normative data from twelve British studies. PLoS ONE.

[10] Caserta D., Graziano A., Monte G.L., Bordi G., Moscarini M. (2013). Heavy metals and placental fetal-maternal barrier: A mini-review on the major concerns. Eur. Rev. Med. Pharmacol. Sci..

[11] Wasserman G.A., Liu X., Factor-Litvak P., Gardner J.M., Graziano J.H. (2008). Developmental impacts of heavy metals and undernutrition. Basic Clin. Pharm. Toxicol..

[12] Zeng X., Xu X., Qin Q., Ye K., Wu W., Huo X. (2019). Heavy metal exposure has adverse effects on the growth and development of preschool children. Environ. Geochem. Health.

[13] Parajuli R.P., Fujiwara T., Umezaki M., Watanabe C. (2013). Association of cord blood levels of lead, arsenic, and zinc with neurodevelopmental indicators in newborns: A birth cohort study in Chitwan Valley. Nepal. Environ. Res..

[14] Wu T., Buck G.M., Mendola P. (2003). Blood lead levels and sexual maturation in U.S. girls: The Third National Health and Nutrition Examination Survey, 1988–1994. Environ. Health Perspect..

[15] Williams P.L., Sergeyev O., Lee M.M., Korrick S.A., Burns J.S., Humblet O., DelPrato J., Revich B., Hauser R. (2010). Blood lead levels and delayed onset of puberty in a longitudinal study of Russian boys. Pediatrics.

[16] NTP (National Toxicology Program, U.S. Department of Health and Human Services) (2012). NTP Monograph Health Effects of Low-Level Lead [WWW Document]. https://ntp.niehs.nih.gov/ntp/ohat/lead/final/monographhealtheffectslowlevellead_newissn_508.pdf.

[17] Nowak-Szczepanska N., Gomula A., Sebastjan A., Ignasiak Z., Koziel S. (2021). Blood lead level and nutritional status indicators in preadolescent Polish schoolchildren. J. Trace Elem. Med. Biol..

[18] Gomula A., Nowak-Szczepanska N., Sebastjan A., Kozieł S.M., Malina R.M., Ignasiak Z. (2022). Age at Menarche in Urban Girls Exposed to Lead in the Copper Basin, Poland. Biology.

[19] Boucher O., Muckle G., Ayotte P., Dewailly E., Jacobson S.W., Jacobson J.L. (2016). Altered fine motor function at school age in Inuit children exposed to PCBs, methylmercury, and lead. Environ. Int..

[20] Fraser S., Muckle G., Despres C. (2006). The relationship between lead exposure, motor function and behaviour in Inuit preschool children. Neurotoxicol. Teratol..

[21] Gbemavo M.C.J., Bouchard M.F. (2021). Concentrations of lead, mercury, selenium, and manganese in blood and hand grip strength among adults living in the United States (nhanes 2011–2014). Toxics.

[22] Taylor C.M., Emond A.M., Lingam R., Golding J. (2018). Prenatal lead, cadmium and mercury exposure and associations with motor skills at age 7 years in a UK observational birth cohort. Environ. Int..

[23] Malina R.M., Little B.B., Buschang P.H., Shephard R.J., Parizkova J. (1991). Estimated body composition and strength of chronically mild-to moderately undernourished boys in southern Mexico. Human Growth, Physical Fitness and Nutrition.

[24] Malina R.M., Pena Reyes M.E., Tan S.K., Little B.B. (2010). Secular change in muscular strength of indigenous rural youth 6–17 years in Oaxaca, southern Mexico: 1968-200. Ann. Hum. Biol..

[25] Ignasiak Z., Sławińska T., Rożek K., Little B.B., Malina R.M. (2006). Lead and growth status of schoolchildren living in the copper basin of south-western Poland: Differential effects on bone growth. Ann. Hum. Biol..

[26] Ignasiak Z., Sławińska T., Rożek K., Malina R., Little B.B. (2007). Blood lead level and physical fitness of schoolchildren in the cooper basin of south-western Poland: Indirect effects through growth stunting. Ann. Hum. Biol..

[27] Bogin B., Varea C., Hermanussen M., Scheffler C. (2018). Human life course biology: A centennial perspective of scholarship on the human pattern of physical growth and its place in human biocultural evolution. Am. J. Phys. Anthr..

[28] Hermaszewski J. (2005). Wpływ Inwestycji na Rozwój Gminy—Doświadczenia Polkowic (English: Impact of Investments on the Development of the Municipality—The Experience of Polkowice).

[29] Martin R., Saller K. (1957). Textbook of Anthropology in a Systematic Presentation with Special Attention to Anthropological Methods.

[30] Parsons P.J., Slavin W. (1993). A rapid Zeeman graphite furnace atomic absorption spectrometric method for the determination of lead in blood. Spectrochim. Acta Part B At. Spectrosc..

[31] Raźniewska G., Trzcinka-Ochocka M. (1993). Monitoring biologiczny olowiu: System i kontrola jakosci. (English: Biological monitoring of lead performance system and quality control). Med. Pr..

[32] Maltin C.A., Delday M.I., Sinclair K.D., Steven J., Sneddon A.A. (2001). Impact of manipulations of myogenesis in utero on the performance of adult skeletal muscle. Reproduction.

[33] Taylor M.P., Isley C.F., Glover J. (2019). Prevalence of childhood lead poisoning and respiratory disease associated with lead smelter emissions. Environ. Int..

[34] Bellinger D.C. (2004). Lead. Pediatrics.

[35] Villareal V., Castro M.J., Riccio C., Sullivan J. (2016). Exposure to lead and other heavy metals: Child development outcomes. Pediatric Neurotoxicology Specialty.

[36] Sławińska T., Ignasiak Z., Little B.B., Malina R.M. (2012). Short-term secular variation in menarche and blood lead concentration in school girls in the Copper Basin of southwestern Poland: 1995 and 2007. Am. J. Hum. Biol..

[37] Stankovic R. (2006). Atrophy of Large Myelinated Motor Axons and Declining Muscle Grip Strength Following Mercury Vapor Inhalation in Mice. Inhal. Toxicol..

[38] Kamel F., Umbach D.M., Munsat T.L., Shefner J.M., Hu H., Sandler D.P. (2002). Lead exposure and amyotrophic lateral sclerosis. Epidemiology.

[39] Praline J., Guennoc A.M., Limousin N., Hallak H., de Toffol B., Corcia P. (2007). ALS and mercury intoxication: A relationship?. Clin. Neurol. Neurosurg..

[40] Malina R.M., Katzmarzyk P.T., Siegel S.R. (1998). Overnutrition, undernutrition and the body mass index: Implications for strength and motor fitness. Med. Sport Sci..

